# Pregnancy-specific responses to COVID-19 are revealed by high-throughput proteomics of human plasma

**DOI:** 10.21203/rs.3.rs-1906806/v1

**Published:** 2022-08-22

**Authors:** Nardhy Gomez-Lopez, Roberto Romero, María Fernanda Escobar, Javier Andres Carvajal, Maria Paula Echavarria, Ludwig L. Albornoz, Daniela Nasner, Derek Miller, Dahiana M. Gallo, Jose Galaz, Marcia Arenas-Hernandez, Gaurav Bhatti, Bogdan Done, Maria Andrea Zambrano, Isabella Ramos, Paula Andrea Fernandez, Leandro Posada, Tinnakorn Chaiworapongsa, Eunjung Jung, Valeria Garcia-Flores, Manaphat Suksai, Francesca Gotsch, Mariachiara Bosco, Nandor Gabor Than, Adi L. Tarca

**Affiliations:** 1 Perinatology Research Branch, Division of Obstetrics and Maternal-Fetal Medicine, Division of Intramural Research, *Eunice Kennedy Shriver* National Institute of Child Health and Human Development, National Institutes of Health, U.S. Department of Health and Human Services (NICHD/NIH/DHHS); Bethesda, Maryland, and Detroit, Michigan, USA; 2 Department of Obstetrics and Gynecology, Wayne State University School of Medicine, Detroit, Michigan, USA; 3 Department of Biochemistry, Microbiology and Immunology, Wayne State University School of Medicine, Detroit, Michigan, USA; 4 Department of Obstetrics and Gynecology, University of Michigan, Ann Arbor, Michigan, USA; 5 Department of Epidemiology and Biostatistics, Michigan State University, East Lansing, Michigan, USA; 6 Center for Molecular Medicine and Genetics, Wayne State University, Detroit, Michigan, USA; 7 Detroit Medical Center, Detroit, Michigan, USA; 8 Department of Obstetrics and Gynecology, Fundacion Valle del Lili, Cali, Colombia; 9 Department of Obstetrics and Gynecology, School of Medicine, Universidad Icesi, Cali, Colombia; 10 Department of Pathology and Laboratory Medicine, Fundación Valle del Lili, Cali, Colombia.; 11 Facultad de Ciencias de la Salud, Universidad Icesi, Cali, Colombia; 12 Centro de Investigaciones Clínicas, Fundación Valle del Lili, Cali, Colombia; 13 Division of Obstetrics and Gynecology, School of Medicine, Faculty of Medicine, Pontificia Universidad Católica de Chile, Santiago, Chile; 14 Systems Biology of Reproduction Research Group, Institute of Enzymology, Research Centre for Natural Sciences, Budapest, Hungary; 15 Maternity Private Clinic of Obstetrics and Gynecology, Budapest, Hungary; 16 First Department of Pathology and Experimental Cancer Research, Semmelweis University, Budapest, Hungary; 17 Department of Computer Science, Wayne State University College of Engineering, Detroit, Michigan, USA

**Keywords:** angiogenesis, circulation, cytokine storm, maternal immune activation, proteome, SARS-CoV-2

## Abstract

Pregnant women are at greater risk of adverse outcomes, including mortality, as well as obstetrical complications resulting from COVID-19. However, pregnancy-specific changes that underlie such worsened outcomes remain unclear. Herein, we profiled the plasma proteome of pregnant and non-pregnant COVID-19 patients and controls and showed alterations that display a dose-response relationship with disease severity; yet, such proteomic perturbations are dampened during pregnancy. In both pregnant and non-pregnant state, the proteome response induced by COVID-19 showed enrichment of mediators implicated in cytokine storm, endothelial dysfunction, and angiogenesis. Shared and pregnancy-specific proteomic changes were identified: pregnant women display a tailored response that may protect the conceptus from heightened inflammation, while non-pregnant individuals display a stronger response to repel infection. Furthermore, the plasma proteome can accurately identify COVID-19 patients, even when asymptomatic or with mild symptoms. This study represents the most comprehensive characterization of the plasma proteome of pregnant and non-pregnant COVID-19 patients.

## INTRODUCTION

Coronavirus disease 2019 (COVID-19) represents an ongoing threat to people around the world^1,2^. To date, over 400 million people have been infected with SARS-CoV-2, the virus responsible for COVID-19^3^, and the death toll has neared 6 million^1^. A growing body of evidence has indicated that pregnant women are at an increased risk of adverse outcomes resulting from COVID-19, ranging from greater rates of admission to the intensive care unit and need for mechanical ventilation to higher risk of death compared to non-pregnant women^4–6^. Moreover, pregnant women with COVID-19 have also been shown to experience more obstetrical complications such as preeclampsia^7,8^, preterm birth^7,8^, and stillbirth^9^. Thus, COVID-19 during pregnancy not only adversely affects the mother, but can also negatively affect quality of life for the offspring^10–18^. Hence, there is an urgent need to understand the pregnancy-driven biological pathways, including immune responses, underlying the increased susceptibility to severe COVID-19 and obstetrical disease.

Upon the onset of the COVID-19 pandemic, multiple investigations have sought to uncover the effects of SARS-CoV-2 infection on maternal, fetal/placental, and neonatal immunity^19–32^. Indeed, we and others have characterized the changes in systemic parameters such as cellular immune responses, virus-specific immunoglobulins, and inflammatory mediators in the maternal peripheral blood and/or cord blood to generate a profile of the maternal-fetal immune responses against SARS-CoV-2 infection^26,33–36^. In particular, comparative studies of pregnant and non-pregnant COVID-19 patients have indicated specific alterations in systemic cytokine levels, peripheral leukocyte subsets, and their activation status^37–40^, providing insights into the mechanisms underlying the increased susceptibility to severe COVID-19 during pregnancy. Such findings are consistent with longitudinal analyses of the general population showing that dynamic changes in systemic cytokines^41,42^, bulk or single-cell gene expression^43^, and leukocyte subsets^43,44^ are characteristic of severe COVID-19. The integration of such omics datasets has also revealed the enrichment of specific cellular processes contributing to disease status and severity, such as inflammation, cell cycle and death, and metabolism^45^. Thus, to further understand the consequences of COVID-19 in pregnant women, the application of high-throughput omics platforms has facilitated the identification of relevant molecules and biological pathways implicated in this disease. Indeed, a recent study profiled over 1,400 proteins in maternal peripheral blood and cord blood and indicated that pregnant women with severe COVID-19 display increased inflammatory and anti-viral signaling compared to asymptomatic pregnant women, while their offspring displayed elevated cytokines associated with T-cell responses and/or inflammasome activation^46^. However, the proteomic dysregulation that distinguishes pregnant from non-pregnant COVID-19 patients has not been elucidated.

Aptamer-based technologies that allow the identification and monitoring of over 1,000 potential target proteins have been utilized to profile the human proteome during normal pregnancy and/or its complications in the maternal plasma^47–51^ and amniotic fluid^52^. Yet, the much-expanded version (4.1) of the SOMAScan platform, which allows measuring of over 7,000 analytes, had not been utilized to study pathology in obstetrics. In this study, we classified pregnant and non-pregnant women according to COVID-19 status and severity, and performed proteomic profiling using the high-throughput SOMAScan platform to determine the differentially affected proteins. Furthermore, we utilized computational approaches to compare and contrast the specific proteins and signaling pathways implicated in COVID-19 between the pregnant and non-pregnant states to enable the development and implementation of predictive models of disease.

## RESULTS

### Characteristics of the study population

#### Pregnant individuals:

**Fig. 1.**
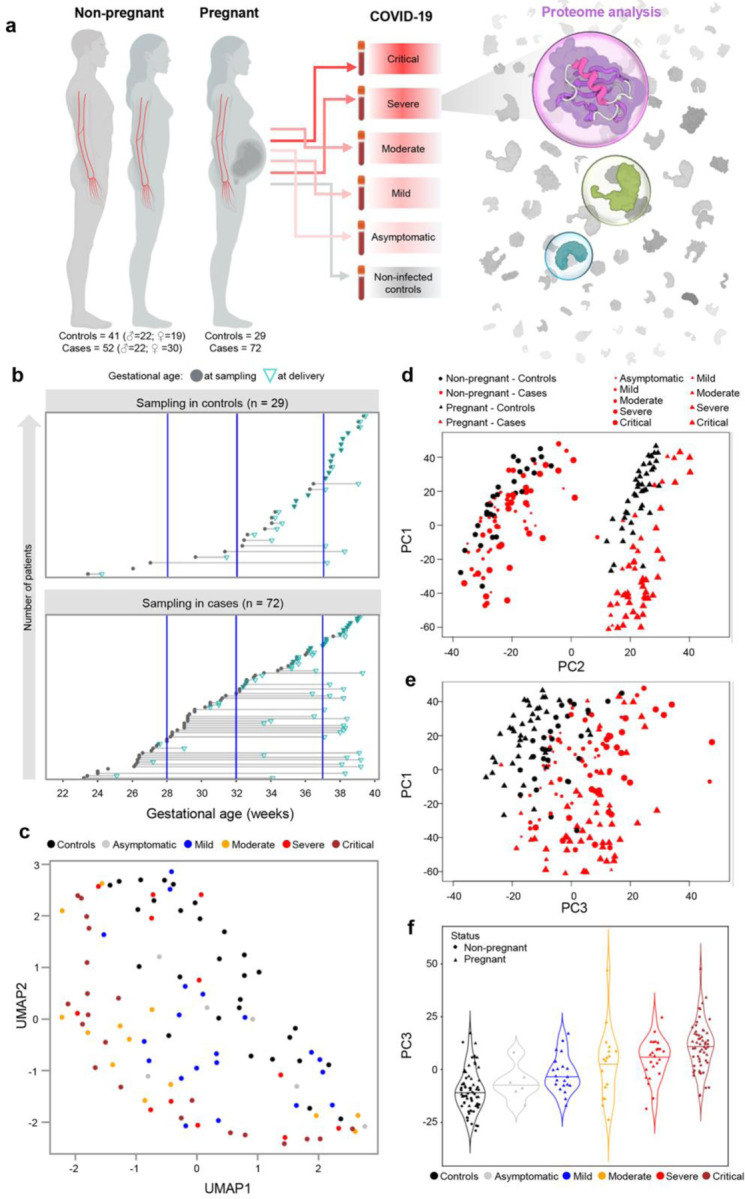
The plasma proteome of COVID-19 patients differs according to disease severity and pregnancy status. **(a)** Illustration of the study design showing the number of non-pregnant controls (n = 41; 22 male, 19 female), non-pregnant COVID-19 cases (n = 52; 22 male, 30 female) pregnant controls (n = 29), and pregnant COVID-19 cases (n = 72) from whom peripheral plasma samples were profiled. **(b)** Gestational age at sampling (grey circle) and at delivery (green triangle) for each pregnant control (upper panel) and case (lower panel). **(c)** UMAP representation of the plasma proteome of pregnant controls and cases. Black = control, grey = asymptomatic case, blue = mild case, yellow = moderate case, red = severe case, brown = critical case. **(d)** Principal component (PC) plot of the plasma proteome of all study samples according to PC1 and PC2. Black = control, red = case. Circle = non-pregnant, triangle = pregnant. Increasing shape size corresponds to increasing COVID-19 severity. **(e)** PC plot representing the relationship between the plasma proteome of all study samples according to PC1 and PC3. **(f)** Violin plot representing the relationship between PC3 and COVID-19 severity among all study samples.

Plasma samples were collected from 101 pregnant women (23.2 – 39.3 weeks of gestation), including those diagnosed with COVID-19 at the time of admission (n = 72) and those who tested negative for SARS-CoV-2 during prenatal care visits (hereafter referred to as pregnant controls; n = 29) (Fig. 1a&b and Table 1). Parameters such as maternal age, BMI, parity, and frequency of chronic hypertension and diagnosis of preeclampsia in the current pregnancy were comparable between the pregnant COVID-19 and control groups (Table 1). Gestational age at delivery was similar between groups; yet, sampling of COVID-19 cases was performed about 5 weeks earlier in gestation than in controls [median weeks (IQR) controls: 36.1 (32.6–37.5) vs. COVID-19: 31.3 (28.1–35.6), p < 0.05] (Table 1), which was considered in the data analysis. Among the pregnant COVID-19 cases, 6 (8%) were asymptomatic, 20 (28%) were mild, 13 (18%) were moderate, 12 (17%) were severe, and 21 (29%) were critically ill according to NIH classification^53^.

#### Non-pregnant individuals:

Plasma samples were also collected from 93 non-pregnant individuals, which included 52 COVID-19 cases and 41 controls (Fig. 1a and Table 1). Among the non-pregnant COVID-19 cases, 1 (2%) was mild, 4 (8%) were moderate, 12 (23%) were severe, and 35 (67%) were critically ill.

### COVID-19 drives shared and unique changes in the plasma proteome in pregnant and non-pregnant individuals that follow a dose response with disease severity

Over 7,000 protein analytes were determined using the SOMAScan v4.1 platform in cases and controls to characterize the plasma proteome responses induced by COVID-19 according to its severity in pregnant and non-pregnant individuals (Fig. 1a). Uniform Manifold Approximation and Projection (UMAP) plots of the proteomic profiles illustrate that patients are clustered according to COVID-19 status and severity in both pregnant (Fig. 1c) and non-pregnant (Supplementary Fig. 1) individuals. It is worth mentioning that, in non-pregnant individuals, the plasma proteome was heavily modulated by COVID-19 status, regardless of sex (Supplementary Fig. 2). Similar to the UMAP depiction, Fig. 1d&e represent an unsupervised projection of high-dimensional proteomic profiles of all controls and COVID-19 patients onto the first three principal components (PC), which can be understood as meta-proteins that are uncorrelated with each other. Notably, pregnancy status represented a source of variability in the proteome, as PC2 values (18% of variance explained) perfectly discriminated between pregnant and non-pregnant individuals (p < 0.001, Fig. 1d). Yet, the host response to COVID-19 represented the primary source of variability in the proteome, as PC1 and PC3 (PC1, 27% of variance explained; PC3, 6% of variance explained) were significantly different between COVID-19 cases and controls, regardless of pregnancy status (p < 0.001 for both, Fig. 1e). The proteomic changes with COVID-19 were larger for non-pregnant than for pregnant women based on both PC1 and PC3 (interaction p < 0.005) (Fig. 1e), which is partly explained by the greater proportions of severe and critically ill cases in the non-pregnant than in the pregnant population. Moreover, we observed a dose-response relationship between PC3 and disease severity, regardless of pregnancy status (p < 0.001 for both linear and quadratic trends, Fig. 1f). Together, these data provide an overview of the plasma proteome in pregnant and non-pregnant individuals infected with SARS-CoV-2, and suggest dramatic changes with infection in a dose-response relationship with disease severity. In addition, these data hint that the host response to SARS-CoV-2 includes shared and unique processes between pregnant and non-pregnant individuals, which we further explore below.

### The plasma proteome response to COVID-19 follows a dose-response relationship with disease severity in pregnant and non-pregnant individuals, yet such a response is dampened in pregnancy

**Fig. 2.**
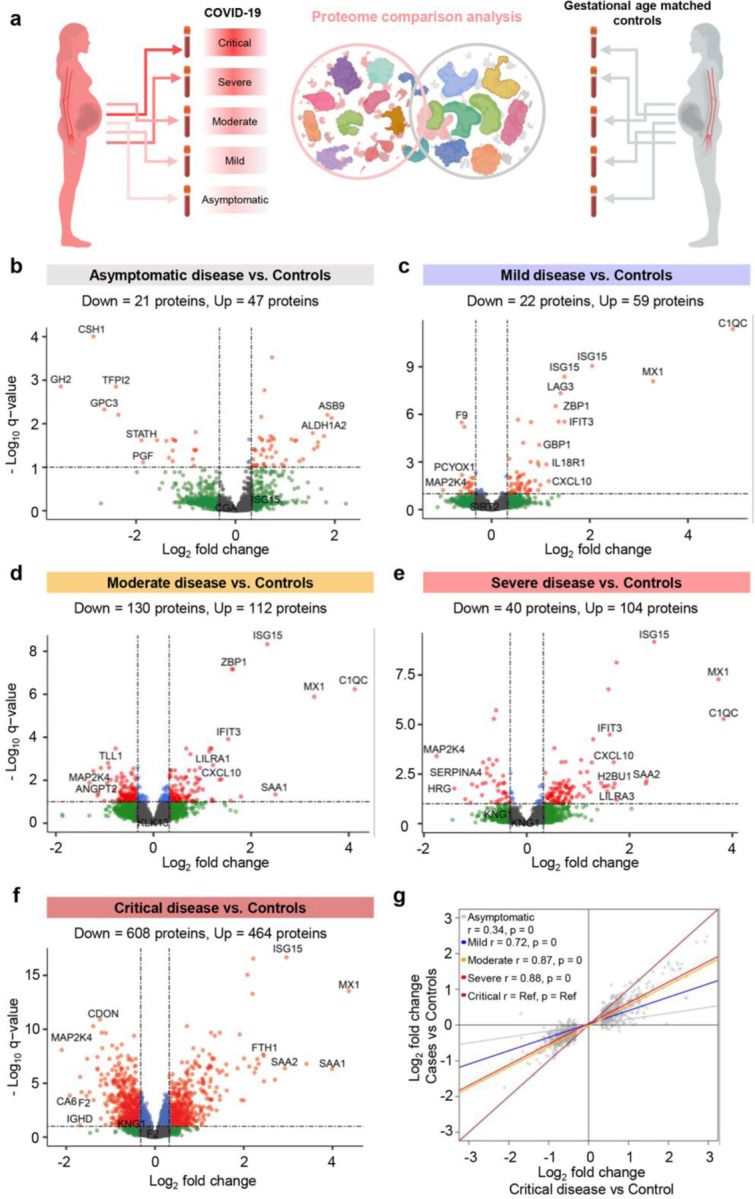
The plasma proteome shows increasing perturbation with COVID-19 severity in pregnancy. **(a)** Graphical representation showing the comparison of plasma proteomes between each classified subset of pregnant COVID-19 cases and controls. **(b)** Volcano plot showing the proteins modulated in asymptomatic COVID-19 cases compared to controls. Red = proteins with q < 0.1 and fold change > 1.25, green = proteins with q ≥ 0.1 and fold change > 1.25, grey = proteins with q ≥ 0.1 and fold change ≤ 1.25, blue = proteins with q <0.1 and fold change ≤ 1.25. **(c)** Volcano plot showing the proteins modulated in mild COVID-19 cases compared to controls. **(d)** Volcano plot showing the proteins modulated in moderate COVID-19 cases compared to controls. **(e)** Volcano plot showing the proteins modulated in severe COVID-19 cases compared to controls. **(f)** Volcano plot showing the proteins modulated in critical COVID-19 cases compared to controls. **(g)** Comparison of the magnitude in proteomic changes among pregnant COVID-19 case subsets, using the comparison between critical cases vs. controls as the reference. Spearman’s correlation and p-value are provided for the asymptomatic vs. control, mild vs. control, moderate vs. control, and severe vs. control contrasts compared to the reference. The proteins included in this analysis (grey dots) are those 1,072 identified as differentially abundant in the comparison between pregnant critically ill cases vs. controls.

Pregnant women have been reported to display heightened susceptibility to severe COVID-19^4–6^. Therefore, we first explored the differential effects of COVID-19 on the maternal proteome compared to control pregnancies according to disease severity (Fig. 2a). When comparing pregnant COVID-19 cases to controls after adjustment for maternal age, BMI, and gestational age at sampling, we identified 68, 81, 242, 144, and 1072 differentially abundant proteins in asymptomatic, mild, moderate, severe, and critically ill cases, respectively (Fig. 2b–f). Given that both disease severity and sample size may affect the number of differentially abundant proteins in specific groups, we next used the protein changes between critically ill patients and controls (1072 proteins) as a reference to compare with the changes observed in the less severe COVID-19 groups (Fig. 2g). The log_2_-transformed fold change of protein abundance between COVID-19 subgroups (i.e., asymptomatic, mild, moderate, and severe) and controls were more attenuated than those between critically ill patients and controls (regression slopes < 1.0) (Fig. 2g). Yet, the magnitude of correlation and the correlation slope followed a dose-response relationship with disease severity, and even asymptomatic patients showed plasma proteomic changes that were significantly correlated to those observed in critically ill patients (r = 0.34 for Asymptomatic vs. Control; r = 0.72 for Mild vs. Control; r = 0.87 for Moderate vs. Control; r = 0.88 for Severe vs. Control; p < 0.001 for all) (Fig. 2g).

**Fig. 3.**
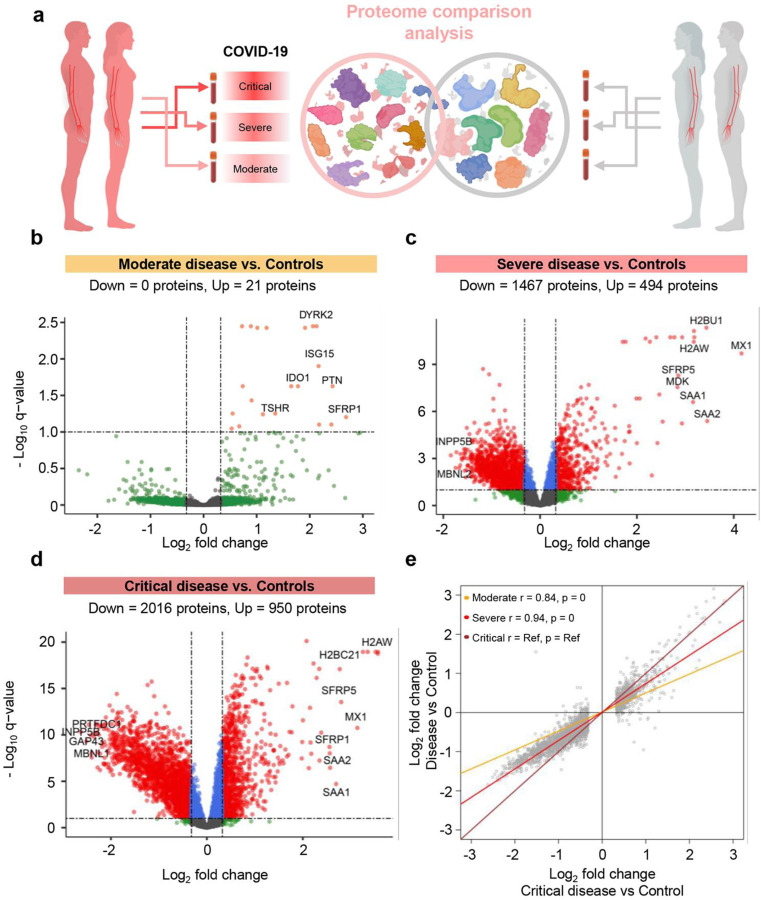
The plasma proteome shows increasing perturbation with COVID-19 severity in non-pregnant individuals. **(a)** Graphical representation showing the comparison of plasma proteomes between each classified subset of non-pregnant COVID-19 cases and controls. **(b)** Volcano plot showing the proteins modulated in moderate COVID-19 cases compared to controls. Red = proteins with q < 0.1 and fold change > 1.25, green = proteins with q ≥ 0.1 and fold change > 1.25, grey = proteins with q ≥ 0.1 and fold change ≤ 1.25, blue = proteins with q < 0.1 and fold change ≤ 1.25. **(c)** Volcano plot showing the proteins modulated in severe COVID-19 cases compared to controls. **(d)** Volcano plot showing the proteins modulated in critical COVID-19 cases compared to controls. **(e)** Comparison of the magnitude in proteomic changes among non-pregnant COVID-19 case subsets, using the comparison between critical cases vs. controls as the reference. Spearman’s correlation and p-value are provided for the moderate vs. control and severe vs. control contrasts compared to the reference. The proteins included in this analysis (grey dots) are those 2,966 identified as differentially abundant in the comparison between non-pregnant critically ill cases vs. controls.

We then performed the same analysis of differential protein abundance in non-pregnant patients (Fig. 3a), and identified 21, 1961, and 2966 differentially abundant proteins in moderate, severe and critically ill cases, respectively (Fig. 3b–d), after adjusting for relevant covariates. Similar to the analysis in pregnant women, the log_2_-transformed fold changes of protein abundance between COVID-19 subgroups and controls were more attenuated than those found between critically ill cases and controls, and followed a dose response with disease severity (r = 0.84 for Moderate vs. Controls; r = 0.94 for Severe vs. Controls; p < 0.001 for both) (Fig. 3e).

**Fig. 4.**
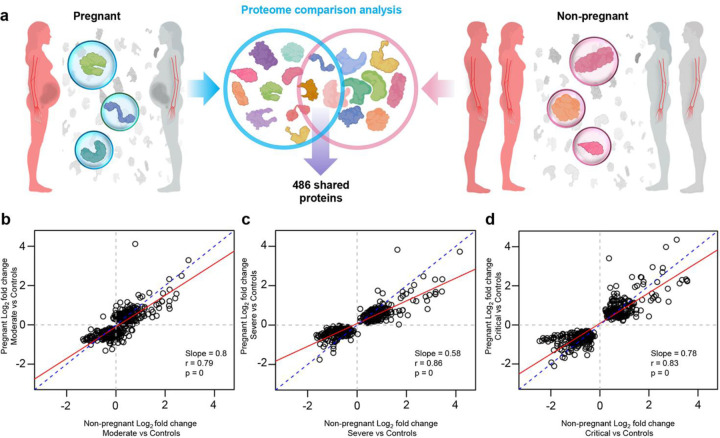
The protein response to COVID-19 is dampened in pregnancy regardless of disease severity. **(a)** Graphical representation showing the comparison of 486 plasma proteins that are modulated in both pregnant COVID-19 cases vs. controls and in non-pregnant COVID-19 cases vs. controls. **(b)** Correlation between the magnitude of proteomic changes in pregnant moderate cases vs. controls and that in non-pregnant moderate cases vs. controls. Slope of the regression line (red line), Spearman’s correlation, and p-value are provided. Dotted blue line represents the parity line. **(c)** Correlation between the magnitude of proteomic changes in pregnant severe cases vs. controls and that in non-pregnant severe cases vs. controls. **(d)** Correlation between the magnitude of proteomic changes in pregnant critical cases vs. controls and that in non-pregnant critical cases vs. controls.

To contrast the magnitude of COVID-19-driven changes in the proteome between pregnant and non-pregnant patients, we then performed correlation analysis based on a core set of 486 proteins with significant and consistent changes in both pregnant and non-pregnant patients (see more details below) (Fig. 4a). By comparing the magnitude of changes between the pregnant and non-pregnant groups, we showed that the magnitude of changes for this set of core proteins were diminished during pregnancy for the same disease severity group, as indicated by the regression line slopes below 1.0 (Fig. 4b–d, p < 0.05 for all).

Together, these results demonstrate that there is perturbation of the plasma proteome in both pregnant and non-pregnant women with COVID-19, and that the magnitude of such changes increases with COVID-19 severity. However, relative to the plasma proteome perturbations observed in non-pregnant individuals, the magnitude of changes with COVID-19 in the pregnant state are attenuated, suggesting a dampened response.

### Shared and distinct changes in the plasma proteome of pregnant and non-pregnant women with COVID-19

**Fig. 5.**
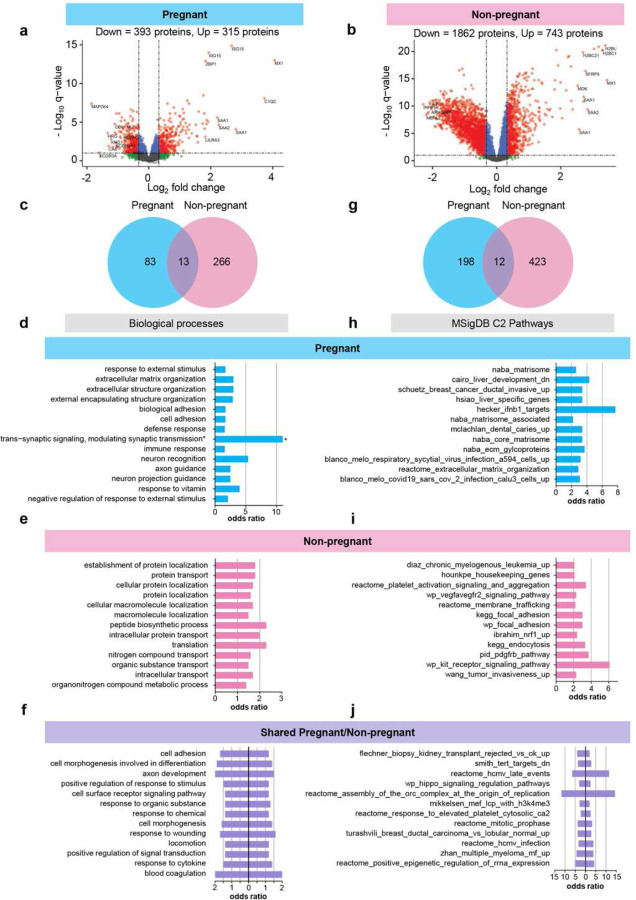
The biological processes and pathways perturbed after COVID-19 differ between pregnant and non-pregnant patients. **(a)** Volcano plot showing the proteins modulated in all pregnant COVID-19 cases compared to controls. Red = proteins with q < 0.1 and fold change > 1.25, green = proteins with q ≥ 0.1 and fold change > 1.25, grey = proteins with q ≥ 0.1 and fold change ≤ 1.25, blue = proteins with q < 0.1 and fold change ≤ 1.25. **(b)** Volcano plot showing the proteins modulated in all non-pregnant COVID-19 cases compared to controls. **(c)** Venn diagram showing the overlap of biological processes enriched among proteins modulated by COVID-19 between pregnant and non-pregnant cases compared to controls. **(d)** Bar plot showing the odds ratios for top biological processes enriched among proteins modulated by COVID-19 in pregnant cases compared to controls. Asterisk indicates odds ratio calculated as “infinite”. **(e)** Bar plot showing the odds ratios for top biological processes enriched among proteins modulated by COVID-19 in non-pregnant cases compared to controls. **(f)** Bar plot showing the odds ratios for top biological processes enriched among proteins modulated by COVID-19 in both pregnant and non-pregnant cases compared to controls. **(g)** Venn diagram showing the overlap of C2 pathways enriched among proteins modulated by COVID-19 in pregnant and non-pregnant cases compared to controls. **(h)** Bar plot showing the odds ratios for top C2 pathways enriched among proteins modulated by COVID-19 in pregnant cases compared to controls. **(i)** Bar plot showing the odds ratios for top C2 pathways enriched among proteins modulated by COVID-19 in non-pregnant cases compared to controls. **(j)** Bar plot showing the odds ratios for top C2 pathways enriched among proteins modulated by COVID-19 in both pregnant and non-pregnant cases compared to controls.

We then sought to further unravel pregnancy-driven differences in the plasma proteomic response to COVID-19 as well as changes that are shared between pregnant and non-pregnant states. First, we identified all proteins that were differentially abundant with COVID-19, which resulted in 708 differentially abundant proteins for pregnant women (Fig. 5a and Supplementary Table 1) and 2,605 for non-pregnant individuals (Fig. 5b and Supplementary Table 2). From these two lists, we identified 486 proteins that were significantly affected by COVID-19 in both pregnant and non-pregnant groups and had similar direction of change (Supplementary Table 2).

Next, we explored the biological processes that were enriched among the entire set of differentially abundant proteins for pregnant (708 proteins) and non-pregnant (2,605 proteins) COVID-19 patients to characterize the differences in host response (Fig. 5c–f). As expected, enriched biological processes in pregnant women with COVID-19 were fewer than those in non-pregnant patients, given the dampened protein response (Fig. 5c and Supplementary Tables 3–4). Consistent with such an observation, pregnant COVID-19 patients showed enrichment of processes related to extracellular matrix, defense response, and immune response (Fig. 5d), whereas those enriched in non-pregnant individuals included protein localization and transport, peptide biosynthesis, and translation (Fig. 5e and Supplementary Tables 3–4). Shared processes were characterized by cell adhesion and immune responses as well as response to wounding and blood coagulation (Fig. 5f).

In addition to biological processes, we also evaluated the enrichment of pathways and gene sets derived from the C2 collection of the MSigDB database (Fig. 5g). Similar to biological processes, pathways enriched in pregnant women with COVID-19 included terms related to extracellular matrix; yet, pathways associated with viral infection or anti-viral defenses were also observed (Fig. 5h and Supplementary Table 5). Enriched pathways in non-pregnant COVID-19 patients included terms related to platelet activation, VEGF, and PDGF (Fig. 5i), while shared pathways included virus- and cancer-related terms (Fig. 5j and Supplementary Table 6).

Together, these data further demonstrate that, although there is a set of common responses to COVID-19 in both pregnant and non-pregnant state, pregnancy-specific changes exist: while non-pregnant women display a stronger proteomic response to fight off infection, pregnant women exhibit a tailored immune proteomic response that may protect the conceptus from unwarranted exposure to inflammation.

### COVID-19 drives distinct angiogenic and inflammatory proteomic changes in pregnant and non-pregnant individuals

**Fig. 6.**
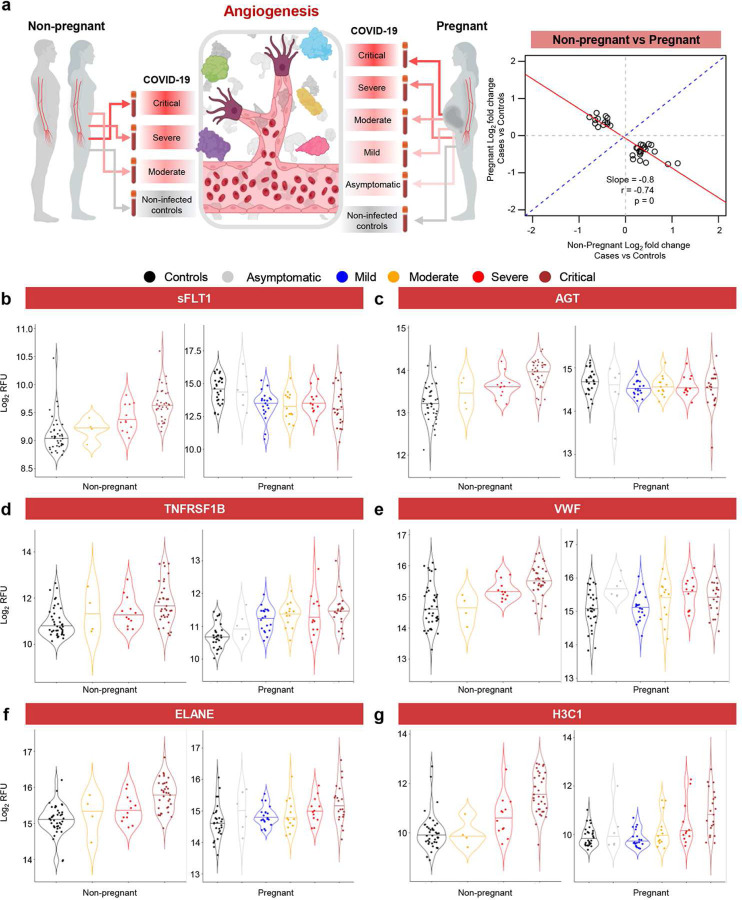
COVID-19 drives distinct angiogenic and inflammatory profiles in pregnant and non-pregnant individuals. **(a) (Left)** Representative diagram illustrating the comparison between pregnant and non-pregnant COVID-19 cases and controls for specific proteins associated with angiogenesis, endothelial dysfunction, and intravascular inflammation. **(Right)** A core set of 33 proteins that are significantly modulated with COVID-19 in opposite directions between pregnant and non-pregnant patients. Note the negative slope and correlation coefficient. **(b-g)** Violin plots showing the modulation of **(b)** sFLT-1, **(c)** AGT, **(d)** TNFRSF1B, **(e)** VWF, **(f)** ELANE, and **(g)** H3C1 levels with COVID-19 severity in non-pregnant and pregnant cases and controls. Black = control, grey = asymptomatic, blue = mild, yellow = moderate, red = severe, brown = critical. RFU = relative fluorescence units.

Given our finding that pregnancy modifies the proteomic response to COVID-19, we further investigated whether any proteins were dysregulated with COVID-19 in opposite directions between pregnant and non-pregnant patients (see Methods). This analysis identified a core set of 33 proteins with opposing direction of change (Fig. 6a) and included proteins related to angiogenesis and wound healing as well as alarmins, cytokines, and growth factors (Table 2). Proteins that decreased with COVID-19 in pregnancy but were increased in non-pregnant cases included vascular endothelial growth factor receptor 1 (VEGF-sR1 or sFLT1) and angiotensinogen (AGT); yet, this could potentially be explained by their already elevated baseline among pregnant patients (Fig. 6b&c and Table 2). Consistent with these findings, proteins that underwent pregnancy-specific regulation with COVID-19 were enriched for biological processes and pathways related to vasodilation, angiogenesis, and regulation of inflammatory response (Supplementary Table 7). A previous report indicated that COVID-19 during pregnancy is characterized by a profile of proteomic factors that is distinct from but overlaps with that observed in preeclampsia^7^, an obstetric syndrome characterized by intravascular inflammation^54^. Therefore, we further evaluated changes in angiogenic or endothelial factors between pregnant and non-pregnant COVID-19 patients. Several factors such as soluble TNF receptor II (TNFRSF1B) and von Willebrand factor (VWF) were found to increase with COVID-19 regardless of pregnancy status (Fig. 6d&e). Notably, neutrophil elastase (ELANE), a neutrophil degranulation factor^55^ as well as a component of neutrophil extracellular traps (NETs)^56^, was elevated in both pregnant and non-pregnant COVID-19 cases (Fig. 6f), as was histone H3.1 (H3C1), another NET component (Fig. 6g). These results provide insight into the unique biological processes in pregnant and non-pregnant individuals: while non-pregnant individuals exhibit increased abundance of angiogenic and inflammatory proteins in the circulation, the proteome of pregnant women hints at a systemic inflammatory response and no increase in anti-angiogenic sFLT-1, which is already elevated in the pregnant state. The latter finding suggests that COVID-19 induces a stereotypical inflammatory response in the maternal circulation that shares pathways with the syndrome of preeclampsia.

### Pregnant women with COVID-19 display a dampened systemic cytokine response

**Fig. 7.**
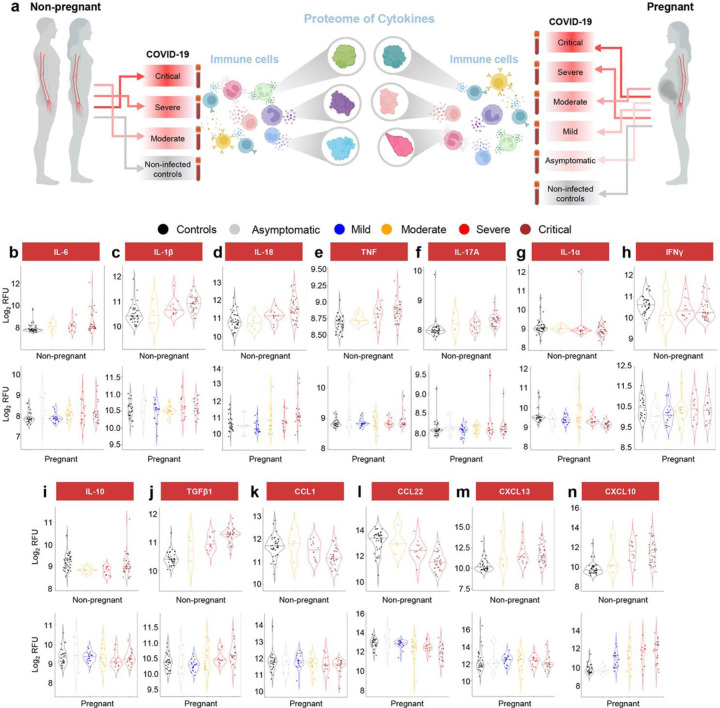
Pregnant women with COVID-19 display a dampened systemic cytokine response. **(a)** Representative diagram illustrating the evaluation and comparison of specific cytokines in the circulation of pregnant and non-pregnant COVID-19 cases and controls. **(b-n)** Violin plots showing the modulation of **(b)** IL-6, **(c)** IL-1β, **(d)** IL-18, **(e)** TNF, **(f)** IL-17A, **(g)** IL-1α, **(h)** IFNγ, **(i)** IL-10, **(j)** TGFβ1, **(k)** CCL1, **(l)** CCL22, **(m)** CXCL13, and **(n)** CXCL10 levels with COVID-19 severity in non-pregnant and pregnant cases and controls. Black = control, grey = asymptomatic, blue = mild, yellow = moderate, red = severe, brown = critical. RFU = relative fluorescence units.

COVID-19 is characterized by a cytokine storm, components of which can display a dose-response with disease severity^41^. Therefore, we next focused on the protein expression changes of specific inflammatory mediators (Fig. 7a). The classical inflammatory cytokines IL-6, IL-1β, and IL-18 were increased in COVID-19 cases compared to controls for both pregnant and non-pregnant patients; yet, the latter two did not reach significance in pregnant women (IL-1β, p = 0.074; IL-18, p = 0.052), likely due to the dampened proteomic response (Fig. 7b–d). Similarly, TNF and IL-17A were upregulated with COVID-19 in non-pregnant patients and only showed a slight tendency to increase during pregnancy (Fig. 7e&f). The alarmin IL-1α was found to be downregulated only in pregnant COVID-19 cases, although a tendency towards the same reduction was observed in non-pregnant patients (Fig. 7g). By contrast, IFNγ was reduced with COVID-19 in non-pregnant individuals but not pregnant patients (Fig. 7h). The anti-inflammatory cytokine IL-10 was downregulated in pregnant and non-pregnant COVID-19 cases (Fig. 7i), whereas TGFβ1 was upregulated in both groups (Fig. 7j). Several chemokines were also found to exhibit differential regulation with COVID-19 in the pregnant and non-pregnant states: CXCL10 and CCL22 were consistently increased or diminished, respectively, in both non-pregnant and pregnant cases; yet, CCL1 was reduced and CXCL13 was increased only in non-pregnant COVID-19 patients, although data from pregnant patients showed similar tendencies (Fig. 7k–n). These findings suggest that COVID-19 induces a cytokine storm in the circulation of both pregnant and non-pregnant individuals; yet, pregnant women display a dampened immune response.

### The plasma proteome can discriminate COVID-19 cases from uninfected controls, even when mild or asymptomatic

**Fig. 8.**
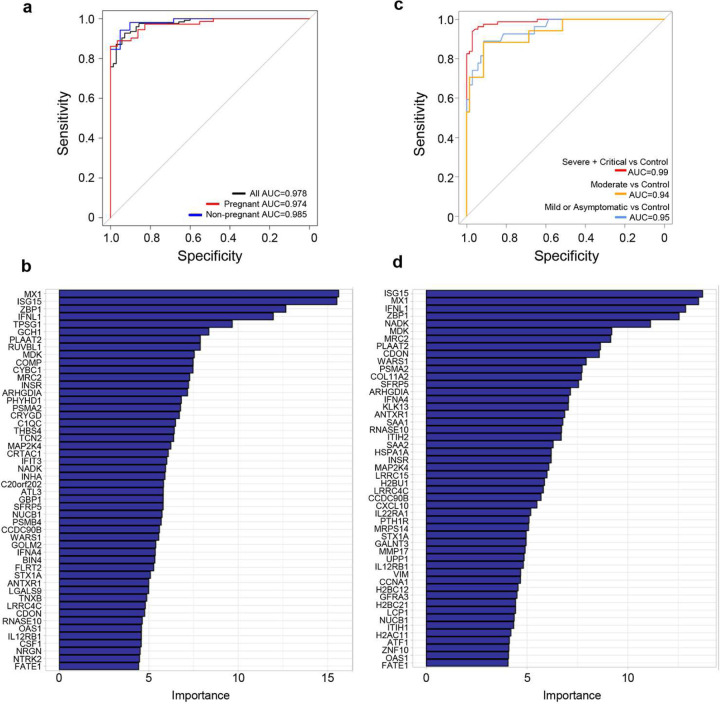
The plasma proteome allows for identification of COVID-19 patients and can distinguish mild and severe disease. **(a)** Receiver operating characteristic (ROC) curves for discrimination of all COVID-19 cases (black curve), only pregnant COVID-19 cases (red curve), and only non-pregnant COVID-19 cases (blue curve) from respective control groups. Area-under-the-curve (AUC) values are shown for each curve. **(b)** Bar plot displaying the relative importance of the top 50 proteomic predictors for identifying all COVID-19 cases. **(c)** ROC curves for discrimination of severe/critical cases from controls (red curve), moderate cases from controls (yellow curve), and asymptomatic/mild cases from controls (blue curve). **(d)** Bar plot displaying the relative importance of the top 50 proteomic predictors for distinguishing severe/critical COVID-19 cases from controls.

Last, we evaluated the ability of the proteomic profiles to discriminate between COVID-19 cases and controls, regardless of pregnancy status. For this purpose, we developed random forests models that included up to 50 proteins and evaluated their accuracy via leave-one-out cross validation (LOOCV). The resulting proteomics model was able to accurately discriminate COVID-19 cases from controls, in the absence of any other inputs (Fig. 8a). The area under the Receiver Operating Characteristic curve (AUC) was 0.978 for the full analysis set, 0.974 for pregnant women, and 0.985 for non-pregnant individuals (Fig. 8a). The relative importance of the proteomic predictors in the random forest model is displayed in Fig. 8b and includes several of the proteins with differential abundance as reported in Supplementary Tables 1–2. When classification models were derived separately based on disease severity, the accuracy to distinguish most severe cases (severe or critical COVID-19) from controls was higher (AUC = 0.99) than the one obtained for discriminating between controls and moderate cases (AUC = 0.94) (Fig. 8c). Of interest, similarly high accuracy was obtained also for distinguishing asymptomatic or mild cases from uninfected controls (AUC=0.95) (Fig. 8c). ISG15, MX1, ZBP1 and IFNL1 were the top four proteins most contributing to the accuracy of random forest models for discriminating all COVID-19 cases from controls, and these proteins were also among the top ones for prediction of severe and critical COVID-19 (Fig. 8d), moderate COVID-19 (Supplementary Fig. 3), and for mild or asymptomatic cases (Supplementary Fig. 4). Together, these data suggest that a shared proteomic signature can discriminate between COVID-19 patients and healthy individuals regardless of pregnancy status, and that disease severity is a driver of classification accuracy.

## DISCUSSION

In this study, we utilized the SOMAScan v4.1 platform to profile over 7,000 protein targets in the peripheral blood of pregnant women and non-pregnant individuals diagnosed with COVID-19, and found that this disease drives changes in their plasma proteomes in a dose-response relation with disease severity. Importantly, we showed that the response to COVID-19 is dampened during pregnancy, regardless of disease severity. Distinct and overlapping proteomic changes were identified in pregnant and non-pregnant COVID-19 patients: pregnant women display a tailored proteomic response, potentially to protect the conceptus from the deleterious effects of inflammation, while non-pregnant women display a stronger response to fight off infection. Moreover, the stereotypical proteomic response induced by COVID-19 in the pregnant and non-pregnant state shows enrichment of mediators implicated in cytokine storm, endothelial dysfunction and angiogenesis; yet, such a response is dampened during pregnancy. Finally, we utilized machine learning to demonstrate that the plasma proteome can be used to discriminate COVID-19 patients from controls, even those who were asymptomatic or had mild symptoms.

The proteomic dysregulations after COVID-19 revealed in our current study are suggestive of a dampened systemic immune response in pregnant women compared to non-pregnant individuals, both in terms of the number of proteins affected and magnitude of changes for proteins implicated in the pregnant and non-pregnant states. This phenomenon could be secondary to physiological changes that occur during pregnancy, such as the reversible thymic involution^57–59^ that impacts T-cell development^60,61^, or could be a primary outcome intended to prevent aberrant immune activation that could threaten pregnancy^62,63^. Immune suppression was originally considered to be a requirement for successful pregnancy, given the immunological puzzle of the mother displaying tolerance towards the semi-allograft fetus for 40 weeks^64^. Rather than complete inertness or unresponsiveness, as proposed by Peter Medawar^64^, pregnancy has since been shown to be a state of selective immune tolerance^65–76^, mediated by homeostatic cells such as regulatory T cells (Tregs)^65–70,73,74,76–86^ and macrophages^81,87–95^. This concept is further supported by studies of women with autoimmune diseases such as systemic lupus erythematosus (SLE), in whom such pregnancy-specific immune adaptations can fail to occur^96,97^, resulting in pregnancy complications^97,98^. Maternal peripheral blood signatures corresponding to IFN responses and immune cell subsets were shown to be significantly modulated throughout normal pregnancy, but less in pregnant SLE patients who experienced complications^97^. Moreover, pertinent to our current findings, the authors of the latter study suggested that the suppression of key immune pathways such as IFN could underlie the higher risk of severe viral infection in pregnant women^97^. Indeed, past and present viral pandemics have provided a large body of evidence showing that specific viruses, such as pandemic influenza viruses, Dengue virus, and coronaviruses, can result in disproportionately high rates of adverse outcomes in pregnant women^99^. Peripheral T and B cells show decreased numbers, greater activation-induced proliferation, and altered phenotypes during pregnancy^100,101^, and such alterations can be further exacerbated by the lymphopenia that characterizes viral infections such as SARS-CoV-2^35,41,43,102^. Moreover, given the demonstrated relationship between pathological maternal T-cell activation and pregnancy complications such as preterm labor^62,63^, it is imperative that maternal adaptive immunity remain under strict control until normal parturition at term^103–106^. Consistently, we recently undertook an *ex vivo* evaluation of peripheral cellular immune responses against SARS-CoV-2 particles and proteins in pregnant and non-pregnant women^30^. We demonstrated a pregnancy-specific reduction of unswitched memory-like and transitional-like B-cell subsets^30^, which is in line with a prior study showing that such reduction of B-cells is associated with COVID-19 severity^107^. Thus, given such deficiencies in peripheral adaptive immunity, pregnant women infected with SARS-CoV-2 may rely more heavily on monocytes, which are also potent contributors to anti-viral host defense^108^. Consistently, monocytes undergo substantial expansion and differentiation in patients with severe COVID-19^109–111^, and we have shown that monocytes from pregnant women appear to undergo accelerated transition and activation in response to SARS-CoV-2 exposure^30^, which is in line with a previous report^39^. Notably, we found that the cytokine profile of peripheral leukocytes was also impacted by pregnancy, as the release of IFN-β and IL-8 in response to SARS-CoV-2 was diminished compared to non-pregnant women^30^. The abovementioned studies, together with our current results, point to a specific dampening of the maternal proteomic response to COVID-19 to protect the fetus from heightened inflammation that could jeopardize pregnancy. This may not be the only mechanism protecting the fetus, as the placenta has also been shown to play a critical role in anti-SARS-CoV-2 defenses^35,112^. The incidence of vertical transmission of SARS-CoV-2 has been shown to be rare, which may be due in part to the minimal co-expression of the canonical viral cell entry mediators *ACE2* and *TMPRSS2* in this organ^19^. Moreover, the placenta exhibits strong anti-viral properties^113,114^, and in women with COVID-19 the placental anti-viral response was shown to include the activation of leukocytes such as T cells, NK cells, and macrophages together with elevated expression of genes related to immune and cytokine signaling, even in the absence of detectable placental infection^35,112^. Thus, the diminished maternal systemic response to SARS-CoV-2 infection may be partially offset by the protective functions of the placenta, thereby preventing a cytokine storm that could damage the fetus while still ensuring a barrier to prevent viral transmission.

Herein, we found that pregnant and non-pregnant patients infected with SARS-CoV-2 exhibit a perturbed proteomic profile characterized by the enhanced release of cytokines and other mediators associated with inflammation, endothelial dysfunction, and angiogenesis. A hallmark of severe COVID-19 is the systemic inflammatory response that includes the exacerbated release of pro-inflammatory immune mediators, termed a cytokine storm^115–119^. Multiple cytokines involved in this response have been proposed as biomarkers of severity and prognosis for COVID-19^42^. Indeed, the peripheral blood concentration of cytokines, including IL-6, is highly correlated with mortality in patients with COVID-19^42,120^, hinting at a key role for IL-6 in the pathophysiology of severe disease. In fact, it has been proposed that IL-6 acts as an amplifier of the inflammatory response triggered by SARS-CoV-2 by activating the NF-κB and STAT3 pathways in non-immune cells such as the vascular endothelium^121^. This concept is in line with the clinical findings showing that the cytokine storm can lead to generalized endothelial dysfunction^117,122^, as was initially suspected early in the pandemic given the rapid emergence of cardiovascular complications in COVID-19 patients^123,124^. The vascular endothelium is an organ with multiple endocrine, paracrine, and autocrine functions, which are required for vascular homeostasis and regulation of vascular tone^125,126^. Therefore, any disruption in these functions can induce vasoconstriction that can progress to ischemia, inflammation, edema, and culminate in a pro-coagulant state^127^. In addition to the indirect induction of endothelial dysfunction due to the host inflammatory response^128,129^, SARS-CoV-2 can also directly interact with the vascular endothelium, as evidenced by viral inclusion structures observed in vascular endothelial cells at multiple body sites in deceased COVID-19 patients^129,130^. SARS-CoV-2 binds to the ACE2 receptor to enter cells, which can impair the activity of the enzyme ACE2 to neutralize angiotensin vasopressors^122,131^. Such impaired ACE2 activity can activate the kallilkrein-bradykinin pathway that results in increased vascular permeability^122,132^. Moreover, the activation of innate immune cells induces the release of toxic mediators such as reactive oxygen species (ROS) and vasoactive substances that can lead to inter-endothelial gaps, thereby further enhancing endothelial permeability^122^. The activation of endothelial cells leads to the production of multiple pro-coagulant factors, such as P-selectin, fibrinogen and Von Willebrand factor (VWF), which initiates the coagulation cascade^122,128^. These processes can also lead to platelet aggregation and the release of other factors such as VEGF, which upregulates the endothelial cell production of tissue factor, the primary stimulator of the coagulation cascade^122,133^, ultimately leading to a pro-thrombotic state. Consistently, herein we showed that, while non-pregnant patients with COVID-19 exhibit angiogenic and inflammatory circulatory profiles, the proteome of pregnant women is characterized by a systemic inflammation without dysregulating the anti-angiogenic factor sFLT-1, which is already elevated in pregnant controls. This factor is a key mediator of the pathophysiology of preeclampsia^134,135^, and is commonly utilized as a biomarker of this obstetrical syndrome^54^. Notably, initial investigations of pregnant women infected with SARS-CoV-2 had revealed the development of a preeclampsia-like syndrome^136,137^. Furthermore, later evidence supported COVID-19 as a risk factor for preeclampsia^8,138^ and indicated a dose-response relationship with disease severity^7^; however, the mechanisms and causality of such an association are still poorly understood^54,138,139^. Our findings revealed that some proteins implicated in inflammatory and angiogenic processes were perturbed in patients with COVID-19, regardless of pregnancy status; yet, there were specific proteins that were only modified by SARS-CoV-2 infection in pregnancy. As preeclampsia is a primarily systemic endothelial-inflammatory obstetrical disease^54,140–144^, our findings support the fact that some perturbed pathways may be shared between COVID-19 and preeclampsia. This is supported by a previous study comparing circulating biomarkers in pregnant women with COVID-19 and those of women with preeclampsia, which demonstrated that preeclampsia and severe COVID-19 display distinct biomarker profiles^145^. Moreover, preeclampsia is a placental disease that is usually resolved after the delivery of this organ^142,146,147^; by contrast, maternal recovery from COVID-19 prior to delivery results in the disappearance of preeclampsia-like symptoms^54,136^. Yet, the similarities between these two disease states are consistent with the placental inflammatory response induced by maternal SARS-CoV-2 infection, even in asymptomatic pregnant women^35,112^. Such inflammation can affect the fetus even in the absence of vertical transmission, as we have demonstrated a mild cytokine response in the cord blood of neonates born to infected mothers^35^. Therefore, it is imperative to follow and evaluate these infants for eventual adverse outcomes, as has been suggested by recent evidence demonstrating neurodevelopmental sequelae at one year of life in children exposed to SARS-CoV-2 *in utero*^18^.

The establishment of biomarkers that allow for the classification and monitoring of COVID-19 outcomes is essential to guide patient management, particularly during pregnancy. In the current study, we demonstrated that the systemic proteome can be utilized to distinguish COVID-19 patients and controls, in the absence of any other patient risk factors. Of importance, the plasma proteome was able to discriminate asymptomatic cases and those with mild symptoms from controls with high accuracy. Our findings are in line with a prior multi-omics investigation that evaluated 1,400 plasma proteins together with single-cell immune features for the classification of non-pregnant COVID-19 patients^148^. In the latter study, such integrated modeling showed value for the distinction of mild, moderate, and severe COVID-19 cases, and identified specific immune features that showed dose-response changes with disease^148^. The use of specific inflammatory mediators in the circulation to characterize COVID-19 was evaluated since the onset of the pandemic, with elevated levels of cytokines (such as IL-6), chemokines, and interferons being reported in cases of severe COVID-19^149,150^ and high systemic levels of IL-6, IL-8, and TNF at the time of hospitalization showing use as biomarkers of disease severity and mortality^42^. In-depth investigations have used longitudinal profiling of COVID-19 patients to identify multiple immune signatures that correlated with different disease trajectories^41^, or utilized proteomic determinations and machine learning to identify 11 host proteins and biomarker combinations that could distinguish and predict COVID-19 outcomes^151^. Interestingly, the presence of neutralizing immunoglobulin G (IgG) autoantibodies against type I interferons has also been shown to represent a likely indicator of severe disease in COVID-19 patients, given that such autoantibodies were absent in most of the individuals with asymptomatic or mild SARS-CoV-2 infection^152^. Together with our current data, these observations point to the value of identifying specific proteomic changes that can serve as biomarkers of COVID-19 severity, particularly during the vulnerable period of pregnancy.

Collectively, the study herein represents the most comprehensive characterization of the plasma proteome of pregnant and non-pregnant individuals diagnosed with COVID-19. The findings reported herein emphasize the distinct immune modulation between the non-pregnant and pregnant states, providing insight into the pathogenesis of COVID-19 as well as a potential explanation for the more severe outcomes observed in pregnant women. Importantly, the unique proteomic profiles observed in pregnant women suggest that the preeclampsia-like syndrome in this population may differ in pathogenesis from the canonical pathways implicated in preeclampsia. Yet, further investigation is required to decipher the unique molecular mechanisms whereby SARS-CoV-2 infection induces a maternal cytokine storm and, more importantly, its effects on the offspring.

## METHODS

### Study design

The study involved profiling of 7,288 proteomic targets in plasma samples collected from pregnant women (n = 101) and from non-pregnant individuals (n = 93). Pregnant patients were enrolled at admission to the labor and delivery unit or at the time of attending the clinical for obstetrical indications or clinical deterioration warranting inpatient management. All patients were screened for COVID-19 according to standard clinical care. Of all controls (patients without COVID-19), those who provided samples within the same gestational age window as cases were retained. Non-pregnant patients were enrolled at time of admission for any medical indication, and all were tested for COVID-19. All analyses accounted for the age and sex of patients as well as the presence of chronic hypertension or high-risk pathology. All patients provided written informed consent, and the use of biological specimens and clinical data for research purposes was approved by the Biomedical Research Ethics Committee of the Fundacion Valle del Lili (Protocol No. 1611), Cali, Colombia. Patients diagnosed with COVID-19 were grouped as asymptomatic, mild, moderate, severe, or critically ill according to NIH classification^53^.

### Plasma proteomics

Maternal plasma protein abundance was determined using the SOMAmer (Slow Off-rate Modified Aptamers) platform and its reagents. This platform allows for the multiplexed quantification of 7,288 analytes corresponding to 6,596 unique protein targets^153–155^. Results herein are presented at the level of analytes, which are also interchangeably referred to as proteins. The experiments were run in batches of up to 85 samples per plate. Briefly, plasma samples were diluted and then incubated with the respective SOMAmer mixes pre-immobilized onto streptavidin-coated beads. The beads were washed to remove all unbound proteins and other matrix constituents. Proteins that remained bound were then tagged using an NHS-biotin reagent. After the labeling reaction, the beads were exposed to an anionic competitor solution that prevents non-specific interactions from reforming after disruption. Pure cognate-SOMAmer complexes and unbound (free) SOMAmer reagents were then released from the streptavidin beads using ultraviolet light that cleaves the photo-cleavable linker used to quantitate proteins. The photo-cleavage eluate, which contains all SOMAmer reagents (some bound to a biotin-labeled protein and some free), was separated from the beads and then incubated with a second streptavidin-coated bead that binds the biotin-labeled proteins and the biotin-labeled protein-SOMAmer complexes. The free SOMAmer reagents were then removed by several washing steps. For the final elution, protein-bound SOMAmer reagents were released from their cognate proteins using denaturing conditions. These SOMAmer reagents were then quantified by hybridization to custom DNA microarrays. The Cyanine-3 signal from the SOMAmer reagent was detected on microarrays^153–155^. Proteomics profiling was performed by Somalogic, Inc. (Boulder, CO, USA).

### Statistical analyses

#### Demographic and clinical characteristics

These data were summarized using numbers and percentages for categorical variables or medians and interquartile range (IQR) for continuous variables. Differences between cases and controls were assessed using the Fisher’s exact test for categorical data and the Wilcoxon test for continuous data. All statistical tests were two tailed and significance was inferred based on p<0.05.

#### Principal component analysis

Protein abundances expressed as relative fluorescence units (RFU) were log_2_-transformed to improve normality. The function *prcomp* in the R statistical language and environment (www.r-project.org) was used to calculate principal components (PC). The top three PC were tested for associations with COVID-19 and pregnancy status using linear models with interaction terms. The dose-response relationship between a given PC and disease severity was assessed using a linear model in which the response variable was the PC and the explanatory variable was an ordered factor with six levels ordered in the sequence: Control, Asymptomatic, Mild, Moderate, Severe, and Critical. This analysis included also pregnancy status, age, and sex of participants as possible confounding variables. All statistical tests were two tailed and significance was inferred based on p<0.05.

#### Differential abundance analysis

The proteomic data preprocessing, including an adaptive normalization by maximum likelihood (ANML) step and a calibration step, were performed by SomaLogic, Inc. The goal of these steps was to make data comparable across samples by calculating plate-specific and analyte-specific scale factors. After log (base 2) transformation, data were compared between pooled COVID-19 cases and controls or compared separately between each disease severity group against controls. When analyzing data from pregnant women, maternal age, body mass index (BMI), and linear and quadratic terms of gestational age were included as co-variates. Analysis of data from non-pregnant subjects included adjustment for age, BMI, and sex of the participant. Models were fit using the *limma* package^156,157^ in R. Protein abundance was considered to have changed significantly with COVID-19 if the fold change was >1.25 and false discovery rate (FDR)^158^ adjusted p-value (q-value) was < 0.1. Spearman correlation coefficients and significance p-values were calculated to determine the similarity of log_2_ fold changes in protein abundance obtained for different COVID-19 severity groups against controls, both within and between pregnant and non-pregnant subjects. Proteins with opposite dysregulation due to COVID-19 between pregnant and non-pregnant groups were defined as proteins being either a) significantly changed with COVID-19 in pregnant women (q < 0.1, fold change > 1.25) but with opposite direction of change in non-pregnant individuals (p < 0.05), or b) significantly changed with COVID-19 in non-pregnant individuals (q < 0.1, fold change > 1.25) but with opposite direction of change in pregnant women (p < 0.05).

#### Gene ontology enrichment analysis

Proteins were mapped using the Entrez gene database^159^ identifiers based on SomaLogic, Inc. protein annotation followed by Gene Ontology^160^. Biological processes over-represented among a given protein set were identified using Fisher’s exact tests. Gene ontology terms with three or more hits and an adjusted enrichment q-value < 0.1 were considered as significantly enriched. The MSigDB collection ^161^ of curated canonical pathways (C2 collection) was also analyzed. Enrichment tests were performed using the *GOStats* package^162^ in Bioconductor enrichment analyses. Biological processes over-represented among a given protein set were identified using Fisher’s exact tests. Gene ontology terms with three or more hits and an adjusted enrichment q-value < 0.1 were considered as significantly enriched. The MSigDB collection ^161^ of curated canonical pathways (C2 collection) was also analyzed. Enrichment tests were performed using the *GOStats* package^162^ in Bioconductor^163^.

#### Predictive model development

To assess the value of plasma proteomic data to discriminate between COVID-19 and controls, we have developed random forest models using up to 50 proteins. The proteins were selected based on their importance to the accuracy of the models using the *randomForest* function in R. The protein selection and random forest model fitting steps were evaluated using leave-one-out cross validation (LOOCV), and receiver operating characteristic curves were derived using the *pROC* package in R.

## Supplementary Material

Supplement 1

## Figures and Tables

**Table 1.** Patient demographics.

Pregnant	Controls (n = 29)	Cases (n = 72)	p-value
Age (years)	29 (25–33)	29 (25–33.2)	0.75
BMI	30.8 (27.2–37.3)	30.3 (27–32.9)	0.27
Nulliparous	75.9% (22/29)	56.9% (41/72)	0.11
Chronic hypertension	13.8% (4/29)	5.6% (4/72)	0.22
Gestational age at sampling (weeks)	36.1 (32.6–37.5)	31.3 (28.1–35.6)	0.003
Gestational age at delivery (weeks)	37.2 (34.6–38)	37.1 (34.9–38.3)	1.00
Preeclampsia	31% (9/29)	18.1% (13/72)	0.19

Non-pregnant	Controls (n = 41)	Cases (n = 52)	p-value
Age (years)	55 (40–63)	59.5 (42.8–69.2)	0.09
BMI	25.9 (24.1–28.4)	27.1 (25–30.8)	0.14
Male	53.7% (22/41)	42.3% (22/52)	0.30
Chronic hypertension	43.9% (18/41)	51.9% (27/52)	0.53

Data are presented as medians with interquartile ranges or as proportions (n/N).

* Missing one datum

** Missing 12 data

## Data Availability

The majority of the data generated in this study are included in the manuscript and/or in the Supplementary Materials. Proteomic data generated in this study are available at the Gene Expression Omnibus (accession number GSE207015). All software and R packages used herein are detailed in the Methods.
