# Supplementary material for: Pregnancy-specific responses to COVID-19 are revealed by high-throughput proteomics of human plasma
Source: Res Sq. 2022 Aug 22:rs.3.rs-1906806. Preprint. [Version 1] doi: 10.21203/rs.3.rs-1906806/v1 (PMC9413722; doi:10.21203/rs.3.rs-1906806/v1)
Supplement: Supplement 1 [file NIHPPRS1906806v1-supplement-1.pdf]

Supplementary Files

This is a list of supplementary files associated with this preprint. Click to download.

- Table2oppositesignproteinstrendpnew.xlsx
- TableS1TotalProteinsPregnantCOVID19.xlsx
- TableS2totalProteinsNonPregnantCOVID19new.xlsx
- TableS3BPinPregResponseCOVID19.xlsx
- TableS4BPinNonPregResponseCOVID19.xlsx
- TableS5C2inPregResponseCOVID19.xlsx
- TableS6C2inNonPregResponseCOVID19.xlsx
- TableS7OffsignCasesControlsBPandC2.xlsx
- SupplementaryFigures.pdf
